# Prediction Model for Bronchopulmonary Dysplasia in Preterm Newborns

**DOI:** 10.3390/children8100886

**Published:** 2021-10-04

**Authors:** Joanna Maria Jassem-Bobowicz, Dagmara Klasa-Mazurkiewicz, Anton Żawrocki, Katarzyna Stefańska, Iwona Domżalska-Popadiuk, Sebastian Kwiatkowski, Krzysztof Preis

**Affiliations:** 1 Department of Neonatology, Medical University of Gdańsk, 80-214 Gdańsk, Poland; idomzal@gumed.edu.pl; 2 Department of Gynaecology and Oncological Gynaecology, Medical University of Gdańsk, 80-214 Gdańsk, Poland; dagmara.klasa-mazurkiewicz@gumed.edu.pl; 3 Department of Pathology, Specialist Hospital in Wejherowo, 84-200 Wejherowo, Poland; zawrocki@gumed.edu.pl; 4 Department of Obstetrics, Medical University of Gdańsk, 80-214 Gdańsk, Poland; kciach@wp.pl (K.S.); krzysztof.preis@gumed.edu.pl (K.P.); 5 Department of Obstetrics and Gynecology, Pomeranian Medical University of Szczecin, 70-111 Szczecin, Poland; kwiatkowskiseba@gmail.com

**Keywords:** bronchopulmonary dysplasia, preterm newborns, gestational age, non-invasive ventilation, respiratory insufficiency, predictive model

## Abstract

OBJECTIVE: To develop a multifactorial model that allows the prediction of bronchopulmonary dysplasia (BPD) in preterm newborns. MATERIALS AND METHODS: A single-center retrospective study of infants born below 32 + 0 weeks gestational age. We created a receiver operating characteristic curve to assess the multifactorial BPD risk and calculate the BPD risk accuracy using the area under the curve (AUC). BPD risk was categorized using a multifactorial predictive model based on the weight of the evidence. RESULTS: Of the 278 analyzed preterm newborns, 127 (46%) developed BPD. The significant risk factors for BPD in the multivariate analysis were gestational age, number of red blood cell concentrate transfusions, number of surfactant administrations, and hemodynamically significant patent ductus arteriosus. The combination of these factors determined the risk of developing BPD, with an AUC value of 0.932. A multifactorial predictive model based on these factors, weighted by their odds ratios, identified four categories of newborns with mean BPD risks of 9%, 59%, 82%, and 100%. CONCLUSION: A multifactorial model based on easily available clinical factors can predict BPD risk in preterm newborns and inform potential preventive measures.

## 1. Introduction

According to the World Health Organization, annually, 15 million children are born preterm [1]. Preterm deliveries are associated with significant morbidity, mortality, high use of health resources, and a high economic burden [2]. A substantial decrease in mortality has been achieved in newborns with extremely low birth weights [3,4]. However, these newborns are prone to numerous severe complications, the most common of which is bronchopulmonary dysplasia (BPD). Several approaches to minimize BPD occurrence have been investigated, including the use of individualized and less invasive therapeutic methods. However, owing to increasing survival rates of very preterm newborns, the number of those developing BPD remains stable [5].

The main feature of BPD is the altered pulmonary development of the alveoli and pulmonary vessels. BPD may have long-term consequences, such as airway obstruction in adulthood, resulting in pulmonary insufficiency [6,7,8,9]. Numerous risk factors for the development of BPD have been reported, including low gestational age [5,10,11], low birth weight [11], need for intubation [7], number of intubations [12], and the duration of MV [11,12,13]. However, the relative impact of these factors and their interrelationships remains controversial.

Several attempts have been made to develop a multifactorial predictive model that allows for the identification of preterm neonates with a high risk of BPD. Most have been unsuccessful due to a low number of cases, a high rate of missing data, and insufficient predictive values [14]. The largest of these studies, although informative, was restricted to extremely premature infants (23–30 weeks’ gestation), which constitute a relatively small fraction of the preterm newborn population [15]. The only reasonably robust model for BPD prediction in neonates born below 32 + 0 weeks of gestational age was developed in the USA and may not be fully applicable to the European health care environment [16]. Additionally, there have been several recent developments in the care of preterm infants, such as prenatal steroid therapy, titrating the amount of oxygen during after-birth resuscitation, or early continuous positive airway pressure, that might have an impact on the risk of BPD. Hence, further research in this area is needed.

We aim to develop a multifactorial model identifying preterm neonates with a high risk of BPD to inform potential preventive measures. Therefore, we quantitatively assessed the impact of particular factors on the risk of BPD in a large consecutive series of preterm neonates, with a focus on postnatal interventions in the delivery room and treatment in the first days of life.

## 2. Materials and Methods

**Figure 1:**
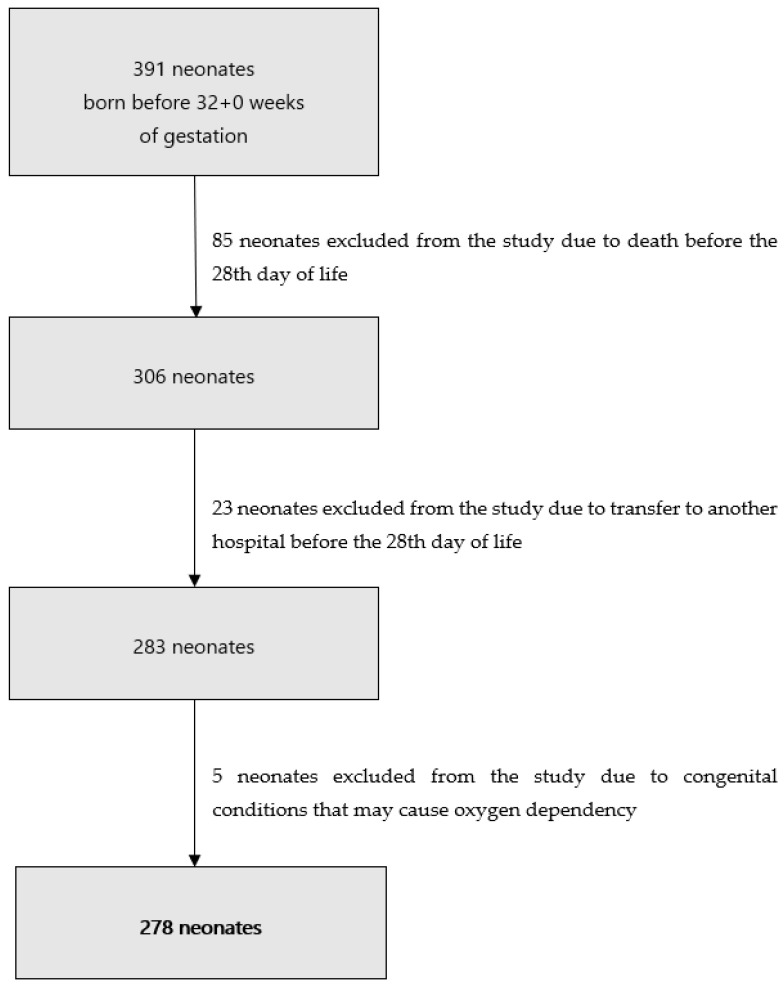
Process of qualification to the study.

This study was based on a retrospective analysis performed in a tertiary referral hospital. The medical records of mothers and neonates with gestational ages below 32 + 0 weeks, born between January 2013 and March 2017, were analyzed. Newborns with congenital pulmonary and heart disease were excluded, as were infants who died or were referred to another hospital before the 28th day of life due to the inability to assess their oxygen requirements on that day. The process of qualification to the study is presented in Figure 1.

BPD was diagnosed according to the definition proposed by Jobe (i.e., requirement of oxygen treatment for at least 28 days after birth) [7]. Disease severity was scored according to the oxygen or ventilatory support requirement at 36 postmenstrual weeks (scored as mild if the infant breathed spontaneously with room air, moderate if the requirement for oxygen was less than 30%, and severe if the newborn needed 30% or more oxygen in the gas mixture or any type of ventilatory support) [7].

Respiratory distress syndrome was defined by the chest X-ray findings using a four-stage scale to assess the intensity of atelectasis. All of the preterm neonates received standard care after birth to achieve stabilization, according to the European Resuscitation Council Guidelines 2010 [17]. RDS was managed following the European Consensus Guidelines on the Management of Neonatal Respiratory Distress Syndrome in Preterm Infants—2013 Update [18]. All of the patients requiring surfactant administration were treated with poractant alfa (Curosurf^®^). Congenital pneumonia was defined as inflammation of the lung parenchyma, as seen on the chest X-ray within the first 24 h of life [19]. Retinopathy of prematurity (ROP) and need for treatment was assessed by examination of the eye fundus by an ophthalmologist, according to the International Classification of Retinopathy of Prematurity [20]. Necrotizing enterocolitis (NEC) was diagnosed based on radiologic signs [21], and stages IA and IB were therefore excluded from the analysis due to a lack of radiologic manifestations. Early-onset and late-onset sepsis were defined as a generalized inflammatory response, with a positive blood culture that was first diagnosed before and after the 72nd hour of life, respectively. Intraventricular hemorrhage (IVH) was defined and staged according to Papile et al.’s definition [22]. Haemodynamically significant patent ductus arteriosus (hsPDA) was defined as open ductus arteriosus that harms the systemic circulation and requires treatment with pharmacotherapy or surgery. All preterm neonates were assessed by a qualified pediatric cardiologist. Hemodynamically and surgical treatment groups were analyzed together due to the low number of cases in each group.

Apart from gestational age and birth weight, small for gestational age (SGA) weight was also analyzed. SGA was defined as birth weight below the 10th percentile according to the Fenton preterm growth charts for girls and boys [23].

Mechanical ventilation (MV) was defined as any type of ventilatory support requiring intubation and artificial ventilation from the ventilator. Positive end-expiratory pressure was defined as ventilatory support delivered by either an InfantFlow^®^ or a Fabian^®^ device in a spontaneously breathing neonate. No distinction was made between continuous positive airway pressure (CPAP) and bilevel positive airway pressure (BiPAP). At the time the research was conducted, high-flow nasal cannulas (HFNC) were not used on preterm newborns at our hospital.

The Apgar score was assessed in the 1st, 2nd or 3rd, 5th, and 10th minutes of life, following the routine assessment performed in our hospital for preterm neonates. The number of newborns requiring mask ventilation, intubation, and ventilation with chest compressions in the delivery room was also assessed.

The data were computed with Statistica 12.5 software (Statsoft Inc., Tulsa, OK, USA). The normality of the distribution was verified with the Shapiro–Wilk test. Since its assumptions were not met, the differences between the independent groups were compared using the Mann–Whitney U-test. The categorical variables were analyzed with the χ2 test, with Pearson’s and Yates’s corrections. The risk factors that were significant in the univariate analysis were subjected to a multivariate logistic regression model. The weight of evidence to express the relative importance of particular factors was calculated using regression coefficients from the proportional hazard regression analysis. Then, a categorical risk-scoring system was established. The numbers provided for the scoring values were rounded to the nearest unit (1.0). These scores were combined to create risk categories and develop a prognostic model. A receiver operating characteristic (ROC) curve was created to assess multifactorial BPD risk. BPD risk accuracy was calculated using the area under the curve (AUC). The factors significant in univariate analysis were applied for the multivariate analysis. Some factors, such as gestational age and birth weight, were strong predictors in univariate analysis but proved to be dependent upon each other in the multivariate analysis. In such a case, two different multivariate analyses with each of those two factors were performed, and the factor with a stronger predictive value was preferred. BPD risk was categorized using a multifactorial predictive model based on the weight of evidence. An alpha error level of <0.05 was considered significant.

## 3. Results

A total of 391 neonates born before 32 + 0 weeks of gestation was identified. Of these, 85 were excluded from the study due to death before day 28 of life, 23 were excluded due to referral to another hospital before the 28th day of life and 5 were excluded due to a congenital condition potentially causing oxygen dependency. Therefore, a total of 278 infants were included in the final analysis (Table 1).

At the 36th week of postmenstrual age, 127/278 newborns (46%) developed BPD, 79 of whom (28%) were categorized as mild, 34 (12%) as moderate, and 13 (5%) as severe. In three patients (1%), the disease severity could not be assessed due to death or referral to another hospital before the 28th day of life.

Respiratory distress syndrome (RDS) occurred in 89% and 72% of newborns who did and did not develop BPD, respectively (*p* < 0.001), including grades 1–2 in 67.7% and 64%, respectively (odds ratio (OR) 2.8; *p* = 0.003), and grades 3–4 in 21% and 8% (OR 6.9; *p* < 0.001), respectively. At least one surfactant dose in both groups was administered in 31.5% and 16.6% of cases, respectively (OR 6.0; *p* < 0.001), and at least two surfactant doses were administered in 54% (range 2–5) and 0%, respectively. Both congenital and late pneumonia were significantly more common in the patients who developed BPD (OR 5.5 and 4.7, respectively; *p* < 0.001 for both). The mean duration of MV in both groups was 13.1 and 0.5 days, respectively (*p* < 0.001), and the mean duration of positive continuous airway pressure was 13.6 and 3.2 days, respectively (*p* = 0.002). The mean duration of oxygen therapy was 51 days in the BPD group and 20.5 days in the no-BPD group (*p* < 0.001).

Small for gestational age (SGA) weight was also under consideration as a risk factor. However, SGA was not selected as a separate category since the number of children with SGA (defined as birth weight below the 10th percentile, according to the Fenton preterm growth charts for girls and boys) in both groups was small: 9 (6%) neonates in the no-BPD group and 10 (7.9%) neonates in the BPD group. Consequently, there was no statistical significance for SGA with the chi-square test (*p* = 0.726).

The newborns with BPD were more frequently affected by other prematurity complications. These included ROP requiring treatment (OR 6.9; *p* < 0.001), NEC (OR 2.7; *p* = 0.008), late-onset sepsis (OR 2.1; *p* = 0.004), IVH (OR 1.5–7.2; *p* = 0.005), and hsPDA (OR 22.0; *p* < 0.001). No significant difference was found for periventricular leukomalacia.

Erythropoietin was administered in 75% and 63% of the newborns who did and did not develop BPD, respectively (no statistically significant difference). In turn, the infants with BPD more frequently underwent red blood cell concentrate (RBC) transfusions (84% and 36%, respectively; *p* < 0.001). Three or more transfusions in both groups were administered to 35% and 0% of the cases, respectively.

The duration of hospital stay in the newborns with BPD was longer (mean 66.1 days vs. 43 days without BPD; *p* < 0.001). A total of 55% and 98% of the cases, respectively, were discharged directly home. The remaining patients were referred to another department or died (three cases with BPD). The mean time of parenteral nutrition in both groups was 36.7 and 18.9 days, respectively (OR 1.2; *p* < 0.001).

The Apgar score in the patients who later developed BPD was lower for all assessment time points (1st minute 5.1 vs. 6.8, p *p* < 0.001; 2nd/3rd minute 5.9 vs. 6.8, *p* = 0.001; 5th minute 6.9 vs. 7.8, p *p* < 0.001; and 10th minute 7.0 vs. 7.7, *p* = 0.020). The resuscitation rates in the groups with and without BPD were 81% and 40%, respectively, and the rates of intubation in the delivery room were 38% and 8%, respectively. The frequency of intubation at any time in both groups was 41% and 8%, respectively (*p* < 0.001), and ventilation accompanied by chest compressions was employed in 7.9% and 3.3% of the cases, respectively (*p* = 0.39).

The significant factors for BPD risk in univariate analysis were resuscitation after birth, use of a T-piece device after birth, Apgar score below 7 at the 5th minute of life, intubation during resuscitation, chorioamnionitis, multiple pregnancies, breech presentation of the fetus, mode of delivery, surfactant replacement therapy, late congenital pneumonia, RBC transfusion and the coexistence of ROP, IVH, NEC, RDS, and hsPDA in addition to late-onset sepsis, late-onset sepsis (LOS), gestational age, birth weight, length of preterm premature rupture of membranes, and duration of parenteral nutrition (Table 2).

We analyzed the number of surfactant administrations in each patient: in the BPD group, 40 children received one dose, 17 received two doses, 7 received three doses, 2 received four doses, and 3 children were administered five doses. In the no-BPD group, 25 patients received one dose; there was not a need to readminister surfactant in that group. We did the same with RBS transfusions: in the BPD group, 32% of children were transfused once, 18% twice, and 35% at least three times, whereas in the no-BPD group, 28% of newborns received one transfusion and 9% two transfusions of RBC.

In the multivariate analysis, four significant factors were chosen to create a predictive model: gestational age, the number of RBC transfusions, the number of surfactant administrations, and hsPDA.

**Figure 2:**
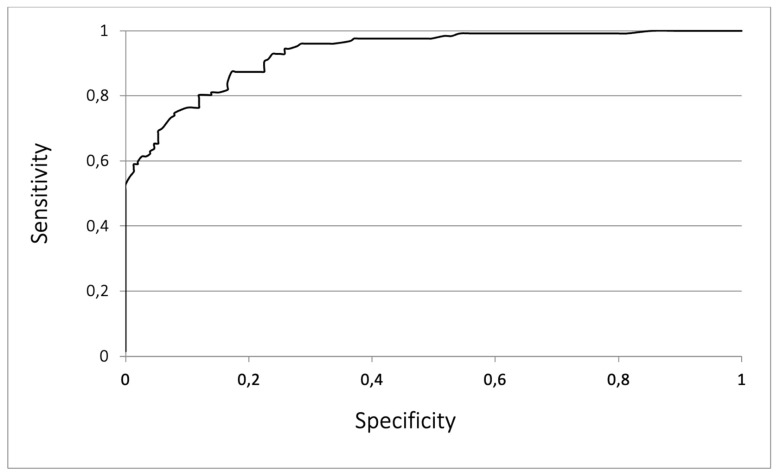
Receiver operating curve for the risk of bronchopulmonary dysplasia including the four-component risk factor model (area under the curve = 0.932).

These four factors were then combined to plot the ROC and to determine the risk of developing BPD, with a resulting AUC value of 0.932 (Figure 2). This ROC curve is a plot of sensitivity on the *y*-axis against specificity on the *x*-axis for a model composed of four factors. The AUC is a summary measure that allows for the prediction of diagnostic accuracy across the spectrum of test values. Next, Weight of Evidence (Statistica^®^) was used to categorize the data. Gestational age, considered a continuous variable, was divided into three age subgroups, with the value homogenously (in a similar pattern) increasing and presenting the most similar ORs within the category. The number of administrations of surfactant and number of RBC transfusions was also categorized, as shown in Table 3, depending on their OR. Later, based on the correlation of particular risk categories with BPD, they were assigned scores corresponding to their significance.

Next, the four factors identified by multivariate analysis were employed to develop a predictive model to assess BPD risk. First, the risk factors were categorized by the number of cases in each subgroup. Then, the significance of each category was assessed based on its OR and assigned a respective number of points for weighting (Table 3).

**Figure 3:**
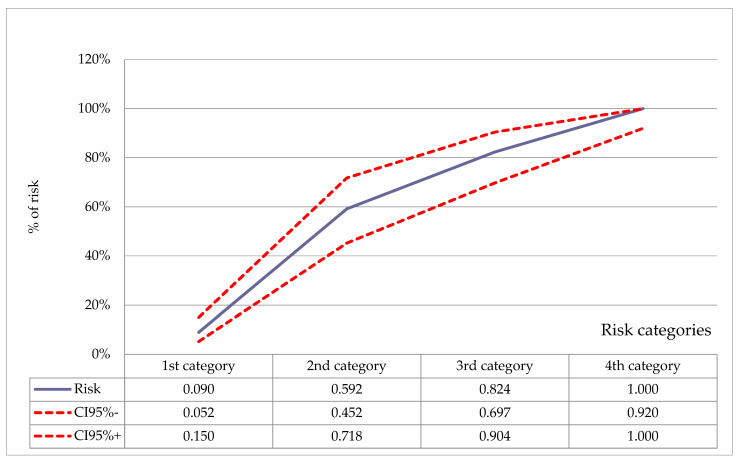
Probability of developing bronchopulmonary dysplasia for particular risk categories.

By summing up the scores in each category, four categories (range 0–17 points), allowing for the prediction of BPD risk, were created as follows (Figure 3):

Low risk (0–4 points): 134 patients; mean risk 9% (95% confidence interval [CI] 5–15%);

Moderately low risk (5–8 points): 49 patients; mean risk 59% (95% CI 45–72%);

Moderately high risk (9–12 points): 51 patients; mean risk 82% (95% CI 73–91%);

High risk (13–17 points): 44 patients; mean risk 100% (95% CI 92–100%).

**Figure 4:**
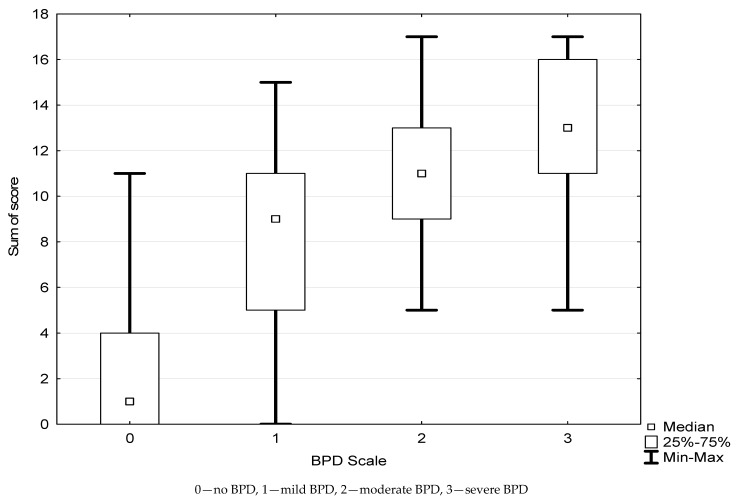
The severity of bronchopulmonary dysplasia in particular risk categories.

Furthermore, we assessed the association between the categories presented in Figure 2 to determine the severity of BPD. The new model (Figure 4) had a significant predictive value for groups with no BPD, mild BPD, moderate BPD, and severe BPD, following the same pattern to sum up the scores for particular risk factors presented in Table 3.

## 4. Discussion

We aimed to develop a multifactorial predictive model for the development of BPD. Such a model might serve as a simple tool to identify newborns with a high risk of this complication and could inform preventive measures for modifiable factors. Finally, it may be an effective aid for communicating with parents and speaking about prognosis.

The study sample included neonates born below 32 + 0 weeks of gestation because BPD rarely develops in more mature newborns. In this study, BPD occurred in 46% of newborns, which is similar to other reports [5]. As expected, and similar to other studies [10,24,25], the main factor associated with BPD development was low gestational age: the mean duration of pregnancy in newborns diagnosed with BPD was 19 days shorter than in those who did not develop this complication. Lower gestational age is inevitably related to lower birth weight. Consequently, this feature was significant only in the univariate analysis but not after adjusting for other significant risk factors. The same was noted for the length of respiratory support and the total number of days of parenteral nutrition. This bias can be avoided in future studies by using more homogenous groups of preterm neonates in terms of gestational age or birth weight.

Chorioamnionitis has long been considered an essential feature associated with the development of BPD [26,27]. However, more recent data indicate that this condition may have a protective role [28], likely due to the more common use of antenatal steroids and surfactant replacement therapy. In our study, chorioamnionitis was associated with BPD only in the univariate analysis.

In newborns who develop BPD, respiratory symptoms typically appear in the first hours of life and are followed by RDS [28]. In our study, this correlation was apparent, and the most severe respiratory symptoms occurred only in the BPD group. Consistent with the findings of Wemhöner et al. [29], the use of surfactants to treat RDS and the number of administrations was also strongly associated with BPD. While the introduction of exogenous surfactants in preterm newborns has decreased the number of complications and improved survival, this has been at the expense of increased BPD risk [30].

In our study, both congenital and late pneumonia were associated with the risk of BPD. Kim et al. [31] showed a correlation between BPD risk and interstitial lung abnormalities found on the 7th day of life. It is still unclear whether BPD is caused by the presence of bacteria in the respiratory tract itself or by the inflammatory response that provokes the disease.

The need for MV or continuous airway pressure is a surrogate for pulmonary function in preterm newborns. In our study, both approaches were used for significantly longer in the patients who developed BPD. Replacing MV with less invasive respiratory support may decrease the risk of this complication [32]. MV mode or type of non-invasive respiratory support should be selected carefully based on clinical presentation, imaging, and blood gas level. Saturation of oxygen target levels should be set at 91–95%, and this parameter should inform the amount of oxygen in the inspiratory mixture of gases [33]. Setting minimal peak pressures, using rapid rates, and starting early therapeutic CPAP in newborns who need intubation and a rapid extubation strategy, along with permissive hypercarbia and hypoxemia, may help reduce the number of ventilation-induced lung injuries [34]. Similar to other studies [11,35], in our study, BPD was accompanied by other complications of prematurity, such as ROP, IVH, hsPDA, NEC, and LOS. In a retrospective analysis from a randomized controlled trial by Potsiurko et al., the markers of hsPDA, that is, the serum NT-proBNP concentration and diameter of PDA, were found to be significant indicators in the first days of life for the development of BPD or death at 36 weeks PMA. In this study, it was noted that higher serum NT-proBNP at 8–9 days of life significantly corresponded with higher FiO2 at this age [36]. These conditions have a similar pathogenesis, and their development in immature newborns is usually provoked by high oxidative stress and the absence of corrective mechanisms.

RBC transfusions in preterm newborns may be associated with an increased risk of some adverse events, particularly NEC [37,38]. However, multiple RBC transfusions may only be surrogates of sickness and not a causative factor. Patel et al. suggested that BPD is related to severe anemia rather than the transfusion itself [39]. In our study, the number of RBC transfusions was strongly correlated with BPD risk. It is, therefore, advisable to consider replacing transfusions with pharmacologic treatment. The association between erythropoietin treatment and the risk of BPD in preterm infants is controversial. Reduced risk of BPD after erythropoietin administration, particularly if initiated within the first four weeks of life, was reported by Rayjada et al. [40]. However, no such association has been shown in randomized studies and meta-analyses [41,42,43,44,45]. In our study, there was no statistically significant difference between the BPD and non-BPD groups in relation to erythropoietin administration.

LOS depends mainly on gestational age. In our series, lower gestational age in the patients with BPD was associated with an almost 1.5 times longer hospitalization than in those who did not develop BPD. Additionally, relatively more newborns with BPD were not discharged directly to the home.

Proper resuscitation and care during the transition period can improve prognosis, particularly by lowering the incidence of IVH, BPD, and accidental hypothermia [46]. In our study, the Apgar score at all assessment points was lower in the newborns who developed BPD, with the highest difference in the first minute. As a consequence, these patients were more often subjected to resuscitation. The proportion of immediate intubation in newborns who did and did not develop BPD was 41% and 8%, respectively. The prevalence of BPD was not related to mask ventilation and intubation directly after birth compared to intubation after a mask ventilation attempt.

A few multifactorial models estimating the risk of BPD in preterm newborns have been proposed. Gursoy et al. [16] developed a model that includes birth weight, gestational age, sex, presence of hsPDA, RDS, hypotension, and IVH, but did not consider each factor’s severity. As in our research, only the hemodynamic significance of PDA was assessed, and not its treatment. Laughon et al. [15], in a study including 3500 preterm neonates, developed a model predicting BPD at different time points during the first month of life. This model included gestational age, type of respiratory support, oxygen pressure, birth weight, sex, race, and origin, but their relevance was not consistent over time. Onland et al. [14] presented a systematic review of 26 predictive models for BPD in preterm newborns. Two main factors increasing the risk of BPD were gestational age and birth weight. The authors emphasized the poor methodological quality of the studies, which was related to the low number of cases, a high rate of missing data, and obsolete statistical methods. Consequently, only 3 out of 26 analyzed studies were recognized as having useful AUC values of more than 0.7 for the ROC curve [14].

Our model includes four factors that proved significant in the multivariate analysis: gestational age, number of RBC transfusions, number of surfactant administrations, and presence of hsPDA. The strength of our model is its simplicity and ease of use in clinical practice. Notably, its AUC value is 0.932, which is remarkably higher than previous models.

The main limitation of our study is the low number of cases in particular subsets, which required them to be merged (an example is patients who required various numbers of RBC transfusions). Second, some infants received transfusions late during their hospital stay; in such cases, the model should be shifted in time. In our study, one of the strongest risk factors was gestational age, which, despite being adjusted for other significant variables, might have confounded other findings. The main shortcoming of our study, however, is the lack of independent external validation of the developed model. Such validation, using appropriate impact analysis, is planned to allow for the clinical implementation of this instrument.

## Figures and Tables

**Table 1:** Patient characteristics.

Variable		BPD Group	No-BPD Group	*p*	OR	95% CI
		*n* = 127	*n* = 151			
Sex	F/M	64/63 (50%/50%)	86/65 (57%/43%)	0.331		
BPD severity	Mild	79 (25%)	N/A			
	Moderate	34 (12%)				
	Severe	13 (5%)				
	data not available	3 (1%)				
Gestational age (weeks)	Mean	27.5	30.3	<0.001	1.2	1.1–1.2
	Median	27.9	30.4			
	Range	<23.4; 31.6>	<27; 32>			
Birth weight (grams)	Mean	1002	1394	<0.001	1.01	1.0–1.01
	Median	970	1385			
	Range	<510; 1990>	<640; 2200>			
SGA		10 (7.8%)	9 (6%)	0.726		
First pregnancy		77 (61%)	80 (53%)	0.246		
Mean mother’s age (years)		29.8 (SD 6.3)	30.7 (SD 5.9)			
Mother’s arterial hypertension	PIH	22 (17%)	23 (15%)	0.799		
	Hypertension before pregnancy	8 (26%)	12 (7.8%)			
Diabetes mellitus	GDMG1	8 (6.3%)	14 (9.3%)	0.731		
	GDMG2	2 (1.6%)	3 (2%)			
	DM1	3 (2.4%)	2 (1.3%)			
Mother’s cervical smear	Physiologic/GBS negative	38 (31%)	57 (38%)	0.772		
	GBS positive	16 (13%)	19 (13%)			
	Escherichia coli	26 (21%)	28 (19%)			
	Ureaplasma urealitycum	17 (14%)	6 (11%)			
	Other, non-physiologic	27 (22%)	31 (21%)			

Legend: BPD—bronchopulmonary dysplasia, F—female, M—male, PIH—pregnancy-induced hypertension, GDMG1—diet-controlled gestational diabetes mellitus, GDMG2—insulin-controlled gestational diabetes mellitus, DM1—diabetes mellitus type 1, GBS—group B streptococcus, SGA—small for gestational age, N/A—not applicable.

**Table 2:** Risk factors for bronchopulmonary dysplasia in univariate analysis.

Factor	BPD/No-PBD Group	*p*	OR
**Gestational age (mean number of weeks)**	**27.5/30.3**	**<0.001**	**0.9**
**Surfactant administration (number of children)**	**69 (54%)/25 (16.6%)**	**<0.001**	**4.7**
**RBC transfusion (number of children)**	**108 (85%)/56 (37%)**	**<0.001**	**3.6**
**Hemodynamically significant PDA**	**33 (26%)/2 (1%**)	**0.012**	**8.8**
Birth weight (mean in grams)	1002/1394	<0.001	1.2
Resuscitation at birth	63 (52%)/34 (23%)	<0.001	5.7
Use of T-piece device at birth	62 (49%)/34 (23%)	<0.001	3.8
Premature rupture of membranes (mean in days)	5.5/2.9	0.1	1.0
Apgar score ≤7 at the 5th minute	102 (80%)/107 (71%)	<0.001	4.3
Intubation in the delivery room	28 (22%)/7 (%)	<0.001	8.0
Presence of intraventricular hemorrhage	24 (19%)/9 (6%)	0.002	3.3
Chorionamnionitis	35 (28%)/20 (13%)	0.005	2.5
Multiple pregnancy			
Twin	39 (31%)/54 (36%)	0.193	0.7
Triplet	1 (1%)/11 (7%)	0.022	0.1
Breech presentation	42 (33%)/33 (22%)	0.049	1.8
Mode of delivery (cesarean section)	66 (52%)/112 (74%)	<0.001	0.4
Late pneumonia	39 (31%)/13 (9%)	<0.001	4.7
Length of stay (mean in days)	66.1/43	<0.001	
Late-onset sepsis	63 (50%)/48 (32%)	0.004	2.1
ROP			
Requiring lasertherapy	31 (24%)/1 (1%)	<0.001	6.9
Not requiring treatment	19 (15%)/15 (10%)	0.033	2.8
NEC	26 (20%)/13 (9%)	0.008	2.7
RDS			
Grade 1 or 2	86 (68%)/96 (64%)	0.003	2.8
Grade 3 or 4	27 (21%)/12 (8%)	<0.001	6.9
Parenteral nutrition (days)	36.7/18.9	<0.001	1.2
Oxygen therapy (days)	51/20.5	<0.001	

Legend: BPD—bronchopulmonary dysplasia, OR—odds risk, RBS—red blood cell, PDA—patent ductus arteriosus, ROP—retinopathy od prematurity, NEC—necrotizing enterocolitis, RDS—respiratory distress syndrome. Factors significant in the following multivariate analysis marked in bold.

**Table 3:** Scores of particular risk factors in the predictive model for bronchopulmonary dysplasia.

Risk Factor	Number of Patients	*p*	OR (95% CI)	Score
Gestational age				
>29 weeks + 5 days	119		1	0
27 weeks + 3 days—29 weeks + 5 days	106	<0.001	13.9 (4.9–39.8)	4
≤27 weeks + 2	53	<0.001	46.3 (15.6–137.4)	8
Surfactant administration				
No	183		1	0
Yes	94	<0.001	5.6 (2.2–14.4)	2
Number of red blood cell transfusions				
0	115		1	0
1	83	0.019	2.9 (1.2–7)	1
>1	80	<0.001	8.7 (3.1–24.6)	3
Hemodynamically significant patent ductus arteriosus				
No	243		1	4
Yes	35	0.007	12.0 (2–73.2)	0

Legend: OR—odds ratio.

## Data Availability

Source data from the study may be achieved from authors upon request.
